# Exploring the potential link between MitoEVs and the immune microenvironment of periodontitis based on machine learning and bioinformatics methods

**DOI:** 10.1186/s12903-024-03912-8

**Published:** 2024-02-02

**Authors:** Haoran Yang, Anna Zhao, Yuxiang Chen, Tingting Cheng, Jianzhong Zhou, Ziliang Li

**Affiliations:** 1 Affiliated Stomatology Hospital of Kunming Medical University, Kunming, Yunnan China; 2 Yunnan Provincial Key Laboratory of Stomatology, Kunming, Yunnan China; 3 Chuxiong Medical College, Chuxiong, Yunnan China

**Keywords:** Mitochondria, Extracellular vesicles, Periodontitis, Immune microenvironment, Machine learning, Bioinformatics

## Abstract

**Background:**

Periodontitis is a chronic inflammatory condition triggered by immune system malfunction. Mitochondrial extracellular vesicles (MitoEVs) are a group of highly heterogeneous extracellular vesicles (EVs) enriched in mitochondrial fractions. The objective of this research was to examine the correlation between MitoEVs and the immune microenvironment of periodontitis.

**Methods:**

Data from MitoCarta 3.0, GeneCards, and GEO databases were utilized to identify differentially expressed MitoEV-related genes (MERGs) and conduct functional enrichment and pathway analyses. The random forest and LASSO algorithms were employed to identify hub MERGs. Infiltration levels of immune cells in periodontitis and healthy groups were estimated using the CIBERSORT algorithm, and phenotypic subgroups of periodontitis based on hub MERG expression levels were explored using a consensus clustering method.

**Results:**

A total of 44 differentially expressed MERGs were identified. The random forest and LASSO algorithms identified 9 hub MERGs (BCL2L11, GLDC, CYP24A1, COQ2, MTPAP, NIPSNAP3A, FAM162A, MYO19, and NDUFS1). ROC curve analysis showed that the hub gene and logistic regression model presented excellent diagnostic and discriminating abilities. Immune infiltration and consensus clustering analysis indicated that hub MERGs were highly correlated with various types of immune cells, and there were significant differences in immune cells and hub MERGs among different periodontitis subtypes.

**Conclusion:**

The periodontitis classification model based on MERGs shows excellent performance and can offer novel perspectives into the pathogenesis of periodontitis. The high correlation between MERGs and various immune cells and the significant differences between immune cells and MERGs in different periodontitis subtypes can clarify the regulatory roles of MitoEVs in the immune microenvironment of periodontitis. Future research should focus on elucidating the functional mechanisms of hub MERGs and exploring potential therapeutic interventions based on these findings.

## Introduction

Periodontitis is a chronic inflammatory disorder caused by plaque microorganisms affecting periodontal supporting tissues [1]. According to the Centers for Disease Control and Prevention (CDC), periodontal disease is the sixth most common disease in humans [2, 3]. Severe periodontitis affects approximately 11% of the global population, making it a global epidemic [4]. During the periodontitis disease process, oral plaque and host immunity interact through a complex communication system. The immune system defends against pathogenic microorganisms and participates in secondary damage to periodontal tissues. However, its specific regulatory mechanisms are not precise [5, 6]. In clinical practice, the graded diagnosis of periodontitis is mainly based on X-ray assessment to detect the presence of alveolar bone resorption. However, X-ray examination cannot accurately show early alveolar bone resorption, which makes it challenging to accomplish the early diagnosis of periodontitis [7]. Thus, how chronic periodontitis can be diagnosed in the early stage of bone destruction and how periodontitis can be ameliorated through immune intervention are urgent problems in the diagnosis and treatment of chronic periodontitis. Further in-depth study of the pathogenesis of periodontitis is the key to solving these problems.

Mitochondria serve as the center of cellular energy metabolism and signaling processes and show critical regulatory functions related to material and energy metabolism, redox state, signaling pathways, cell survival and apoptosis. Govindaraj et al. have shown that mitochondrial function is abnormal in patients with periodontitis [8]. Significant mitochondrial fragmentation and malformation are observed in fibroblasts undergoing apoptosis in periodontitis patients [9]. Sun et al. found that mitochondrial oxidative stress plays a vital role in the aggravation of periodontitis by diabetes mellitus [10]. Based on the findings of these investigations, the maintenance of mitochondrial quality control appears to play a pivotal role in the pathogenesis of periodontitis. However, the mechanisms regulating mitochondrial function in periodontitis are unknown, which hinders the study of therapeutic strategies targeting mitochondria for periodontitis. On the other hand, extracellular vesicles (EVs) are vesicle-like bodies that are produced by cells and secreted into the extracellular space [11]. EVs were initially believed to be biologically inactive and nonfunctional particles. However, the significance of EVs has been elucidated in recent years, showcasing their vital involvement in fundamental biological mechanisms such as immune modulation, angiogenesis, tissue regeneration, and regulation of the tumor microenvironment [10, 12–14]. EVs can be secreted by many cells and carry DNA, RNA, lipids, metabolites, and various proteins. EVs can mediate long-distance communication between different tissues and cells to modulate the behavior of target cells, which offers new research avenues for diagnosing and treating many intractable illnesses. Periodontal ligament stem cells (PDLSCs), gingival MSCs, bone marrow MSCs, osteoblasts, osteoclasts, and periodontal pathogenic bacteria in periodontal tissues can release different components of EVs [15–17]. Compared with EVs from other tissues in the body, EVs from periodontal tissues are easier to isolate [18], and the detection of EVs in gingival sulcus fluid is expected to enable the early diagnosis and dynamic monitoring of chronic periodontitis [19]. In addition, studies have shown that immunotherapy for chronic periodontitis can be performed using exogenous EVs; EVs present in periodontal pathogenic bacteria have been employed to develop vaccines to prevent periodontal diseases [20]. However, research on the role of EVs in chronic periodontitis is still in the primary stage, and there is a need for recognized molecular markers.

**Fig. 1:**
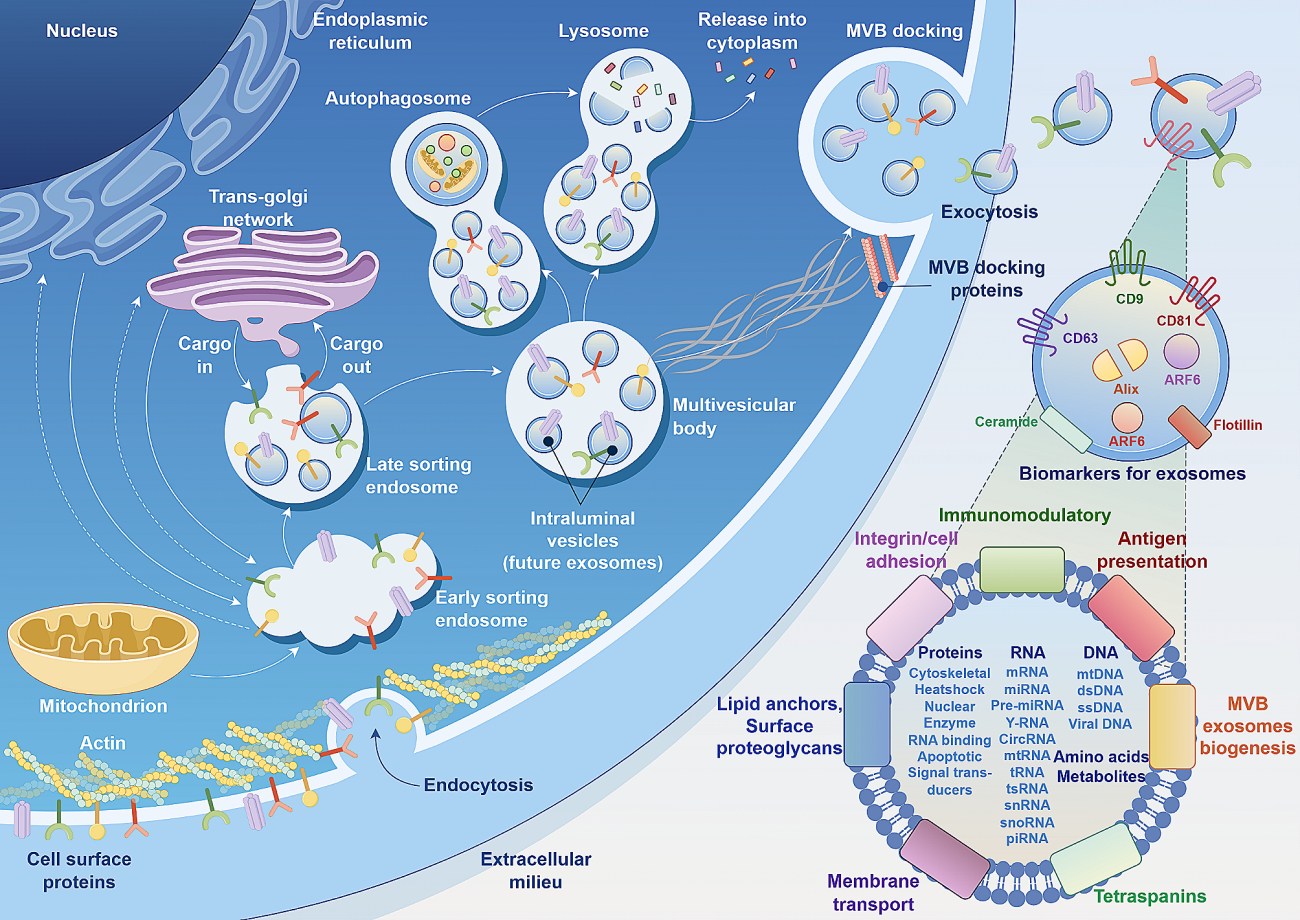
Occurrence and characterization of MitoEVs: an example of mitochondria-rich exosomes. Mitochondria-derived exosomes are formed through the accumulation of proteins, nucleic acids, and lipids synthesized by the endoplasmic reticulum, where the Golgi apparatus mediates the formation of outgrowth structures characterized by vesicles. Subsequently, these vesicles merge with the plasma membrane and are then liberated into the extracellular milieu, culminating in the formation of MitoEVs

Many recent studies have revealed that mitochondrial components, including mitochondrial DNA, mitochondrial RNA, mitochondrial proteins, and mitochondrial fragments, are widely present in specific EV subpopulations (Fig. 1). These EVs regulate the metabolic state and cellular phenotype of recipient cells, and their contents or composition varies with the progression of various diseases, such as tumors, psychiatric diseases and autoimmune diseases, and aging [21]. In addition, EV-mediated mitochondrial transfer has been shown to exert beneficial effects such as repairing the metabolic functions of damaged recipient cells [22]. In summary, it is reasonable to hypothesize that MitoEVs may be used to diagnose or treat periodontitis. However, the potential link between MitoEVs and periodontitis remains to be elucidated.

**Fig. 2:**
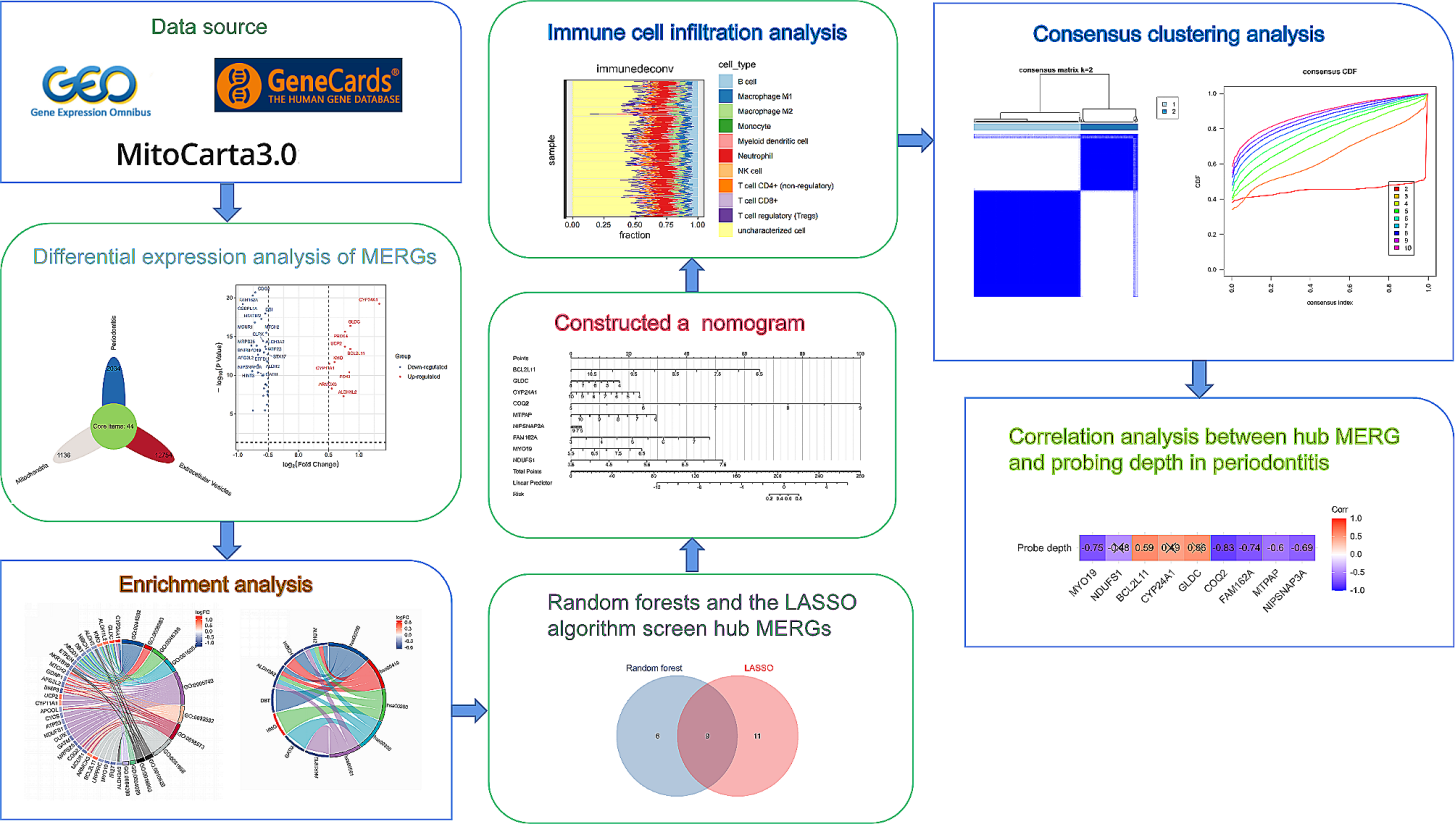
Flowchart of this study

In this investigation, machine learning techniques were employed to develop a classification model. Notably, the model exhibited high accuracy in distinguishing periodontitis gingival tissues from healthy counterparts. Furthermore, an analysis encompassing immune infiltration and consensus clustering was conducted. Subsequently, the correlation between the hub MERGs and probing depth in patients with periodontitis was investigated utilizing the Pearson correlation coefficient. Overall, the study demonstrated the robust classification and diagnostic abilities of the MERG-based model. Additionally, by exploring the relationship between hub MERGs and immune cells and investigating immune cell variations among distinct subtypes, insights into the regulatory role of MitoEVs in the immune microenvironment of periodontitis were gained (Fig. 2).

## Methods

### Data download and processing

The 247 samples included in this study came from the GSE10334 dataset. They included 183 diseased samples and 64 healthy samples, all from gingival tissues from both periodontal healthy and disease states. The externally validated dataset was GSE16134. The GSE106090 dataset, which contains the probing depth for periodontitis, was also included in this study. The aforementioned datasets were acquired from the Gene expression omnibus (GEO) database (http://www.ncbi.nlm.nih.gov/geo/). Mitochondria-related genes were obtained from the MitoCarta3.0 database (https://www.broadinstitute.org/). Extracellular vesicle-related genes were obtained from the GeneCards database (https://www.genecards.org/).

### Differential expression analysis

The “limma” software package was employed for the analysis of MERGs. Genes were considered differentially expressed if they exhibited a |log2FC (fold change)| > 0.5 and a P value < 0.05. Visualization of the results pertaining to the differentially expressed MERGs was carried out utilizing the “ggplots” and “pheatmap” packages.

### Enrichment analysis

To understand the functions of genes and identify pathways with important functions, we conducted enrichment analysis, which encompasses the kyoto encyclopedia of genes and genomes (KEGG) and gene ontology (GO) analyses [23]. The main GO categories are molecular function (MF), biological process (BP), and cellular component (CC) [24]. The analysis was performed using the R software package “clusterProfiler” [25].

### Screening of hub MERGs

The LASSO algorithm is a regression algorithm that can adjust model parameters, reduce model complexity, and avoid overfitting to improve model generalization. A random forest is a classifier consisting of multiple decision trees that allows the random selection of a subset of features. To implement the LASSO algorithm, we utilized the “glmnet” R package, considering the minimum lambda value as the designated threshold [26]. To execute the random forest algorithm, the “random forest” R package was employed, with a threshold set for the relative importance score to be greater than 2 [27].

### Construction of a nomogram

A diagnostic nomogram of hub MERGs was constructed to predict the occurrence of periodontitis. To evaluate the reliability of the developed nomogram, we employed calibration curves. The diagnostic performance of the nomogram was assessed by constructing the receiver operating characteristic (ROC) curve and calculating the area under the curve (AUC). The ROC curve was generated using the “pROC” R package [28], while the “rms” R package was utilized to construct the nomogram [29].

### Immune cell infiltration analysis

Utilizing periodontitis gene expression data, we assessed immune cell infiltration levels to investigate the immune microenvironment of periodontitis. This analysis was conducted using the Web Classification tool (http://CIBERSORT.stanford.edu/), with a reference set comprised of twenty-two immune cell genes (LM22) [30]. The resulting immune cell infiltration data were visualized using the “ggplots” and “pheatmap” packages. Spearman correlation coefficient analysis was employed to calculate correlations between different immune cells, and the visual representation of these correlations was generated using the “corrplot” package. Furthermore, Pearson correlation coefficient analysis was utilized to investigate the correlation between the abundance of immune cells and the expression of hub MERGs [31].

### Consensus clustering analysis

Using the “ConsensusClusterPlus” package in R software, consensus cluster analysis of periodontitis patients was performed based on the hub MERGs [32]. Then, the different MitoEV-related periodontitis subtypes were constructed, and the differences in hub MERGs among different types of periodontitis were compared. The LASSO regression algorithm screened critical immune cells. Then, we proceeded to compare the variances in immune cell populations among diverse subtypes of periodontitis.

### Correlation analysis between MERGs and probing depth in periodontitis

Periodontal probing is the most crucial test in the diagnosis of periodontitis. The GSE106090 dataset contains data on the probing depth of healthy samples for each periodontitis sample. The analysis entailed utilizing the Pearson correlation coefficient to investigate the correlation between the expression levels of hub MERGs and the probing depth of periodontitis. This evaluation has the potential to facilitate clinical diagnosis and treatment approaches.

## Results

### Results of differentially expressed periodontitis MERGs

**Fig. 3:**
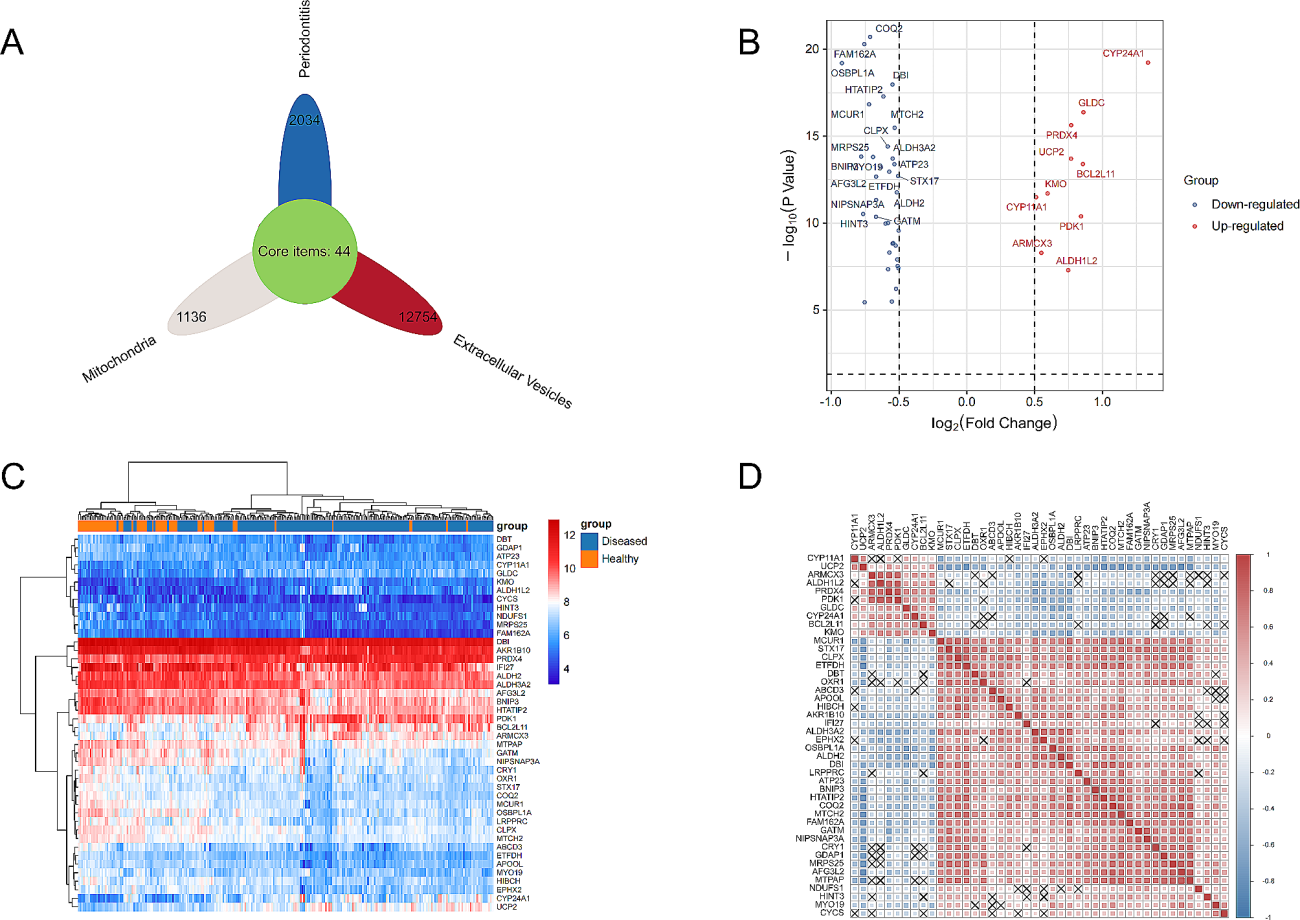
Identification of differentially expressed MERGs. **A** The petal plot identifies 44 MERGs. **B** Volcano plot showing differentially expressed MERGs between periodontitis and healthy gingival tissues. **C** Expression levels of 44 MERGs in gingival samples. Rows and columns denote MERGs and samples, respectively. **D** Correlation heatmap showing correlations among 44 MERGs, where “×” indicates no correlation

**Fig. 4:**
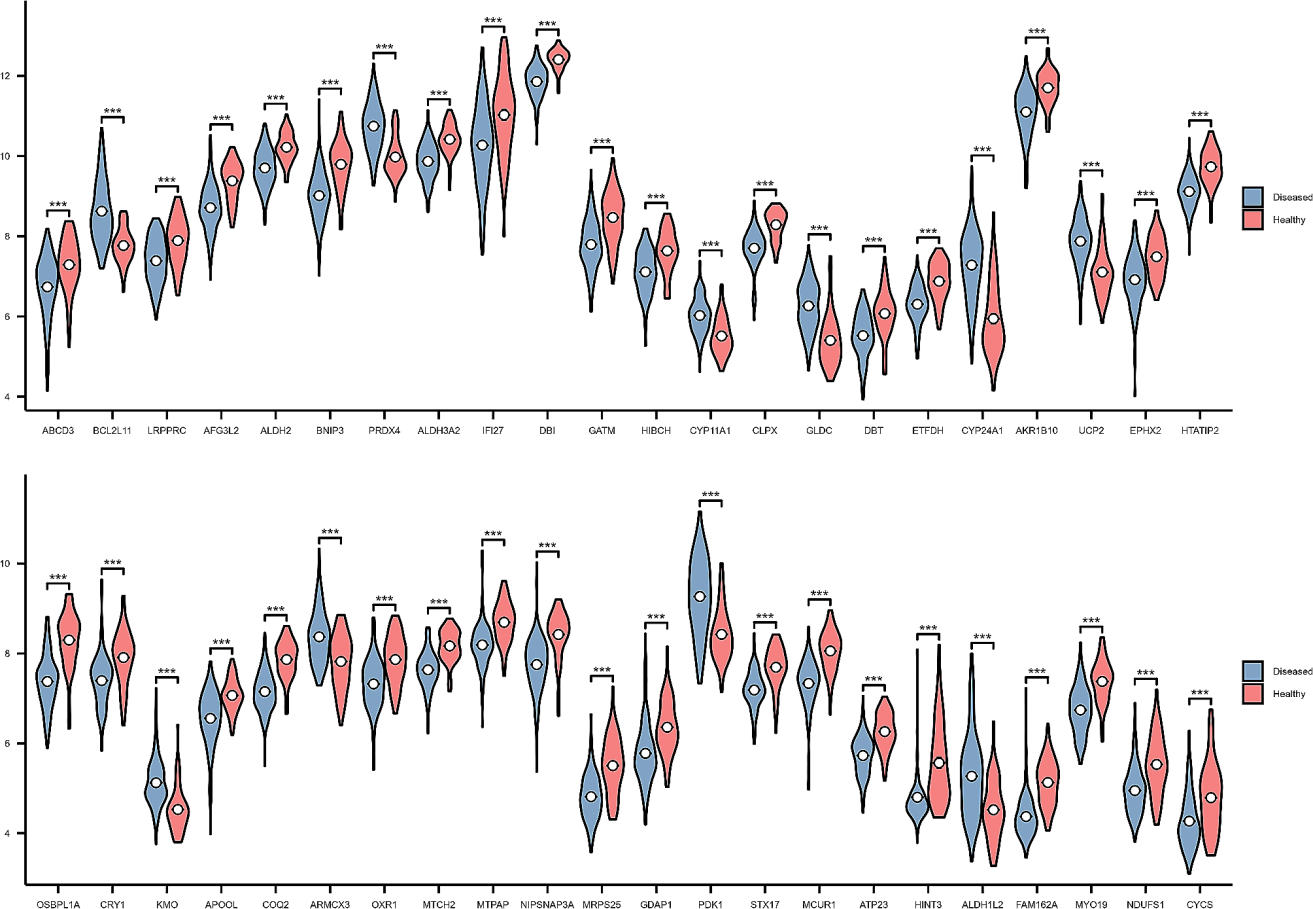
Violin plot showing all 44 MERGs with significantly different expression levels. MERGs, MERGs; ***P < 0.001

A total of forty-four differentially expressed MERGs were discovered, as illustrated in Fig. 3A. Among them, ten genes were found to be upregulated, while thirty-four genes were downregulated, as depicted in Fig. 3B. The heatmap exhibited in Fig. 3C visually represents the variations in gene expression levels between the disease and healthy groups, while the correlation heatmap presented in Fig. 3D displays the interrelationships among the forty-four genes. Moreover, based on Fig. 4, a significant distinction was observed in the expression levels of these forty-four genes between the healthy and disease groups.

### Enrichment analysis results

**Fig. 5:**
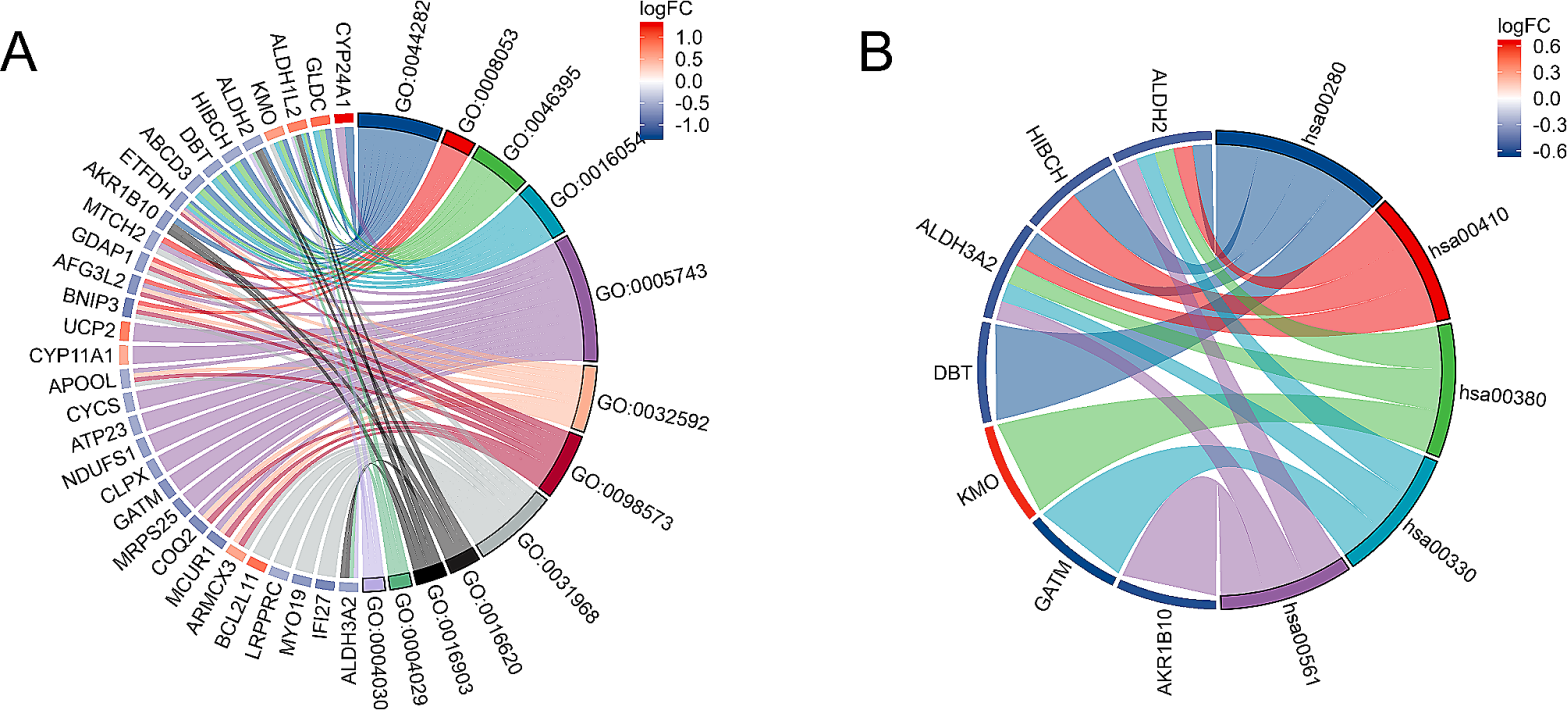
Results of GO and KEGG pathway analyses of MERGs. **A** GO enrichment results. **B** KEGG pathway enrichment results

Enrichment analysis revealed that within the BP category, the MERGs exhibited significant enrichment in various processes, including small molecule catabolic processes, mitochondrial fusion, carboxylic acid catabolic processes, organic acid catabolic processes, and alcohol metabolic processes. In the CC category, enrichment was observed primarily in the mitochondrial inner membrane, integral and intrinsic components of the mitochondrial membrane, organelle outer membrane, and outer membrane. Within the MF category, enrichment was predominantly identified in oxidoreductase activity, aldehyde dehydrogenase (NAD+) activity, aldehyde dehydrogenase [NAD(P)+] activity, and electron transfer activity (Fig. 5A). Furthermore, KEGG pathway analysis revealed significant enrichment of MERGs in pathways associated with valine degradation, leucine degradation, isoleucine degradation, beta-alanine metabolism, and tryptophan metabolism (Fig. 5B).

### Results of machine learning screening for hub MERGs

**Fig. 6:**
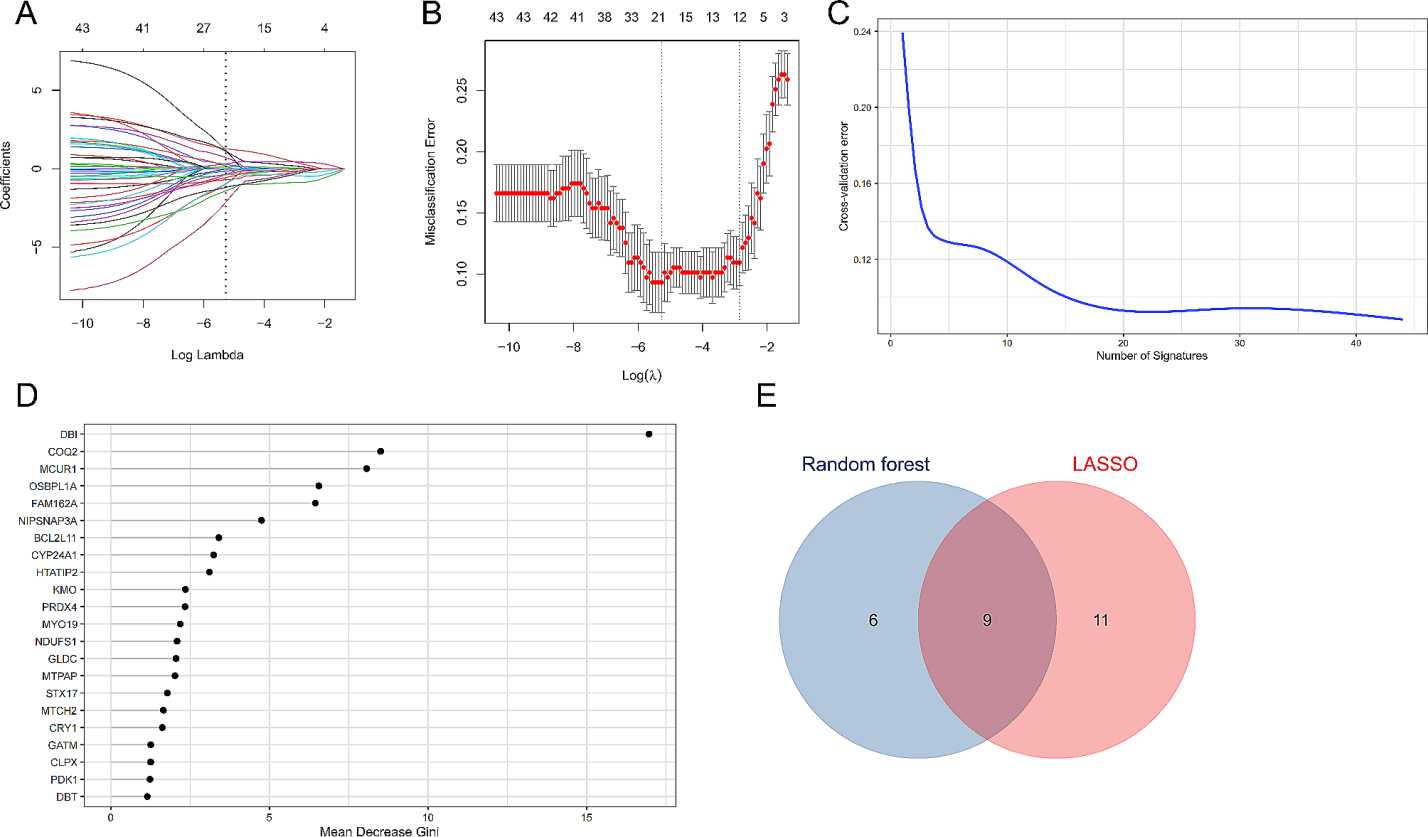
Screening for hub MERGs. **A** LASSO coefficient profiles of the 44 MERGs. **B** Tenfold cross-validation was performed to identify the optimal tuning parameter (λ). **C** Graphical representation depicting the impact of the decision tree count on the model error. **D** The Gini coefficient method random forest classifier was used to filter results. **E** Venn diagram of the shared genes between the random forest and LASSO algorithm datasets

The LASSO regression and random forest algorithms were used to screen hub MERGs. A total of 20 MERGs were screened using the LASSO algorithm (Fig. 6A, B), and 15 were screened using the random forest algorithm (Fig. 6C, D). Finally, we obtained 9 hub MERGs from the intersection of the two algorithms; these MERGs included BCL2L11, GLDC, CYP24A1, COQ2, MTPAP, NIPSNAP3A, FAM162A, MYO19, and NDUFS1 (Fig. 6E).

### Results of the constructed diagnostic nomogram

**Fig. 7:**
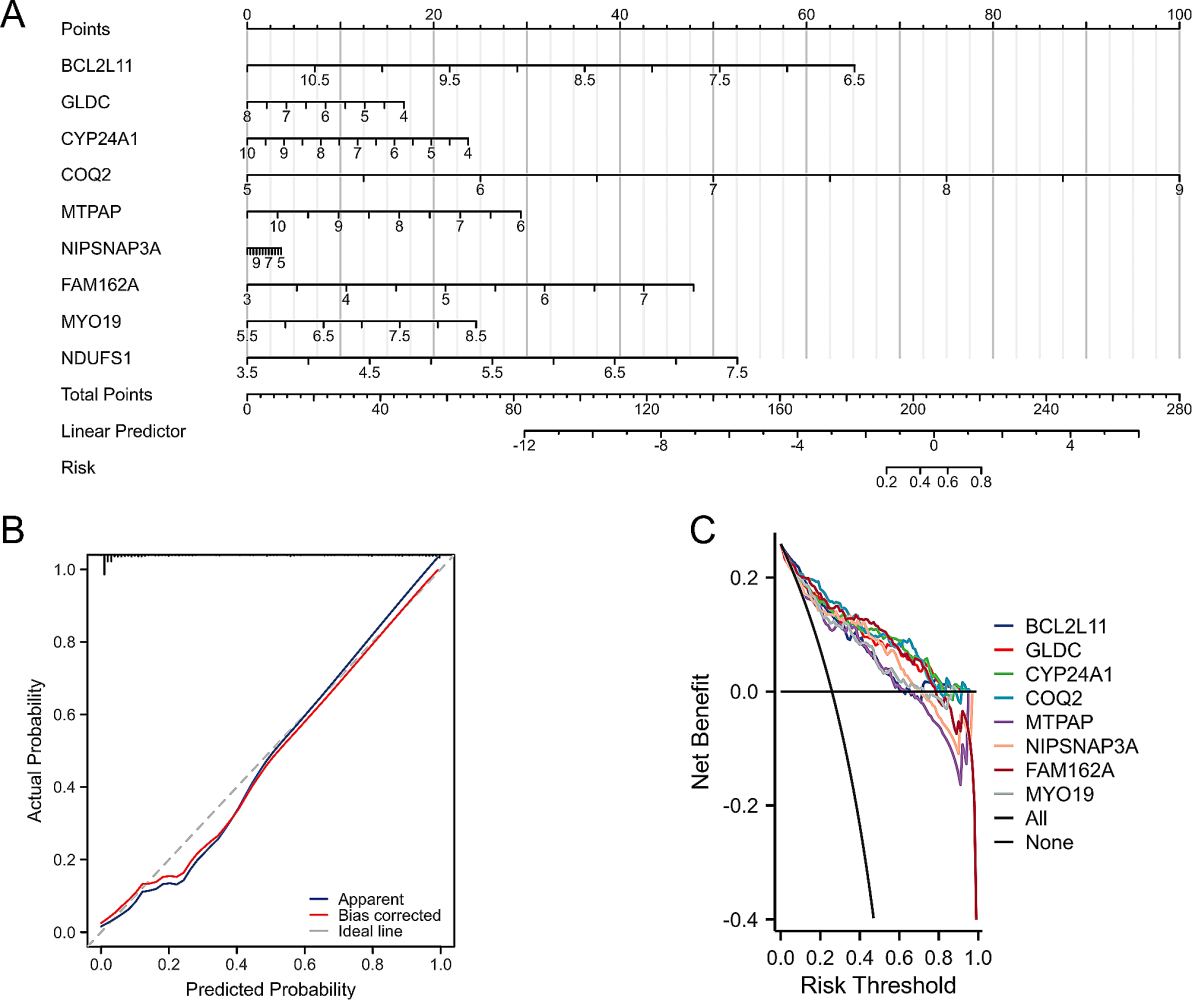
**A** Nomogram. **B** Calibration curve. **C** Decision curve

To help clinicians diagnose periodontitis, we constructed a diagnostic nomogram based on the expression scores of hub MERGs in the GSE10334 dataset. The nomogram showed that periodontitis patients presented a significantly increased risk as the total score increased (Fig. 7A). Calibration curve analysis showed that the nomogram could accurately predict the occurrence of periodontitis (Fig. 7B). Decision curve analysis showed that as the threshold probability increased, the model had a higher level of net payoff (Fig. 7C).

### Validation of classification models using internal and external datasets

**Fig. 8:**
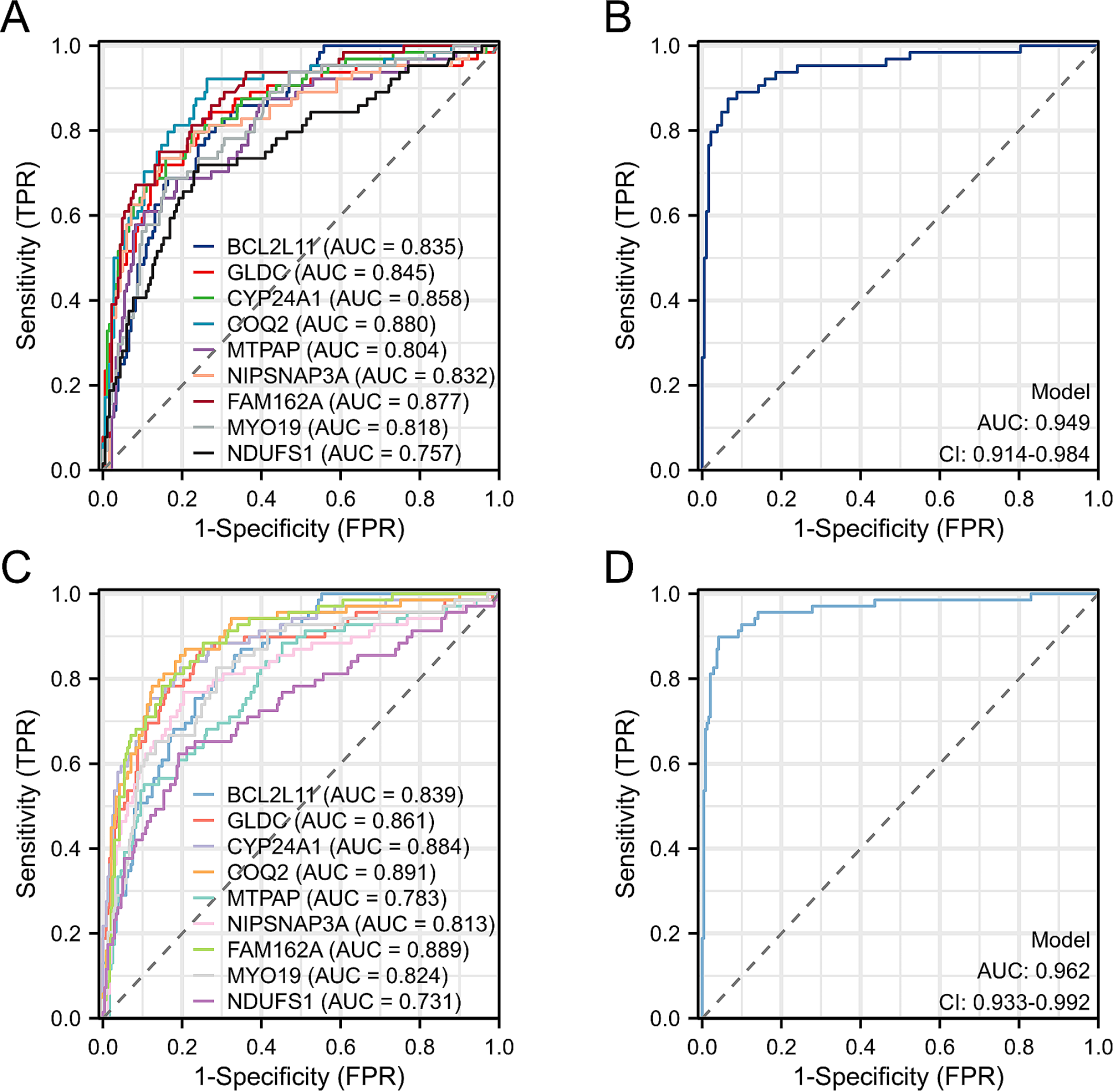
**A** ROC curves of MERGs in the GSE10334 dataset. **B** ROC curves of classification models in the GSE10334 dataset. **C** ROC curves of MERGs in the GSE16134 dataset. **D** ROC curves of classification models in the GSE16134 dataset

The accuracy of the model was assessed using the AUC values for the internal dataset GSE10334 and external dataset GSE16134. In the GSE10334 dataset, the AUC values of BCL2L11, GLDC, CYP24A1, COQ2, MTPAP, NIPSNAP3A, FAM162A, MYO19, and NDUFS1 were greater than 0.7 (Fig. 8A), and the AUC value of the classification model was 0.949 (Fig. 8B). In the GSE16134 dataset, the AUC values of BCL2L11, GLDC, CYP24A1, COQ2, MTPAP, NIPSNAP3A, FAM162A, MYO19, and NDUFS1 were greater than 0.7 (Fig. 8C), and the AUC value of the classification model was 0.962 (Fig. 8D). The results show that the classification model presents excellent classification and discrimination abilities.

### Results of immune infiltration analysis

**Fig. 9:**
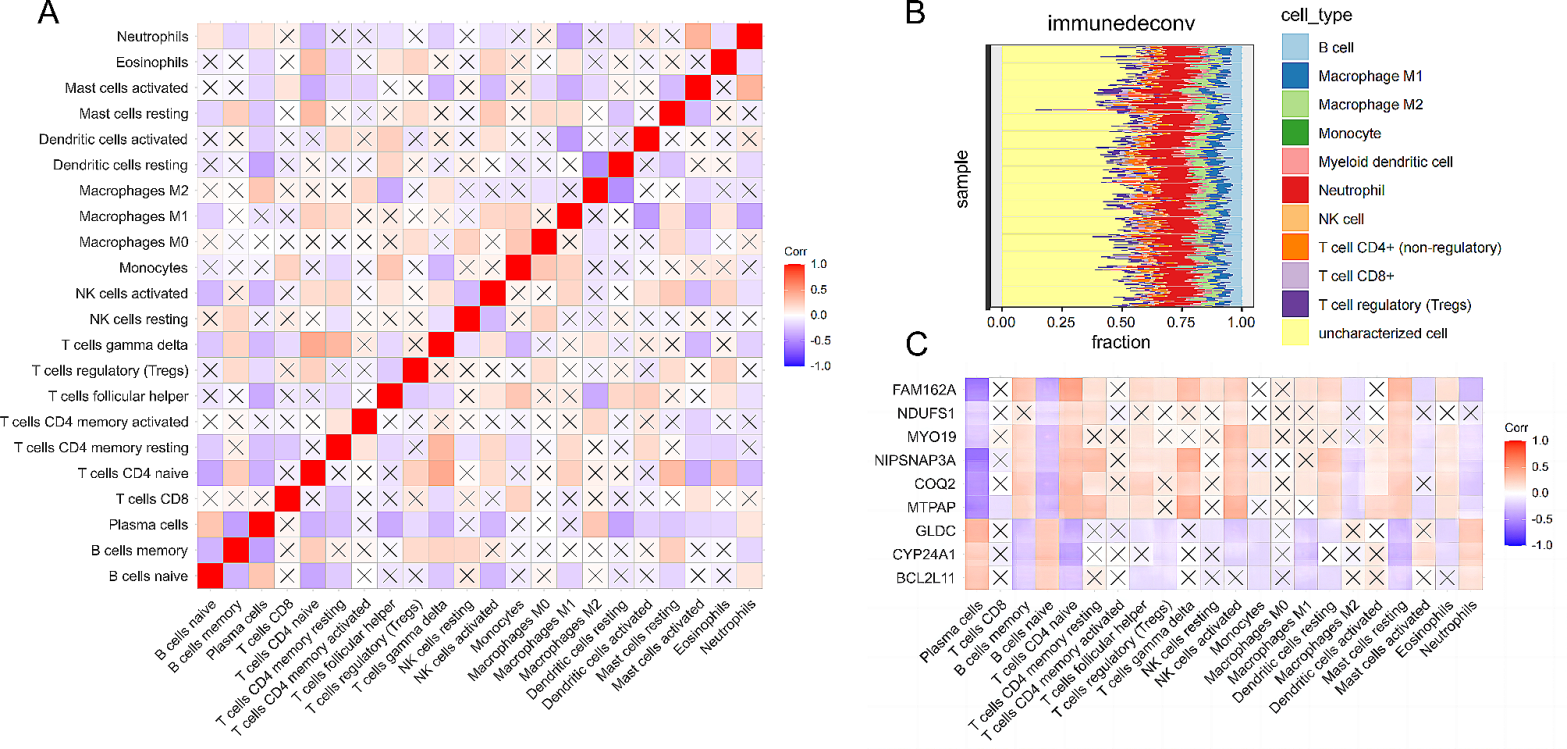
Differential immune cell infiltration in periodontitis and its correlation with hub MERGs. **A** Pearson correlation analysis between periodontitis immune cells. All correlation coefficients not indicating correlations are marked with “×”. **B** Infiltration ratio of immune cells for each periodontitis sample. **C** Pearson correlation analysis between hub MERGs and immune cells. All correlation coefficients not indicating correlations are marked with “×”

**Fig. 10:**
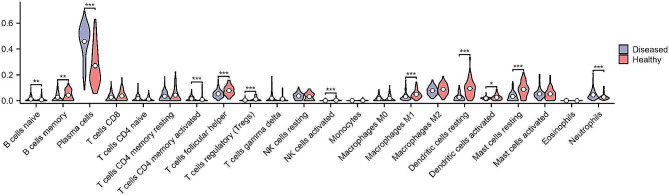
Violin plot showing all 12 types of immune cells with significantly different expression levels in periodontitis; *P < 0.05, **P < 0.01, ***P < 0.001

By analyzing the association between hub MERG expression and immune cell infiltration in periodontitis, we conducted a comprehensive investigation into the potential molecular mechanisms through which hub MERGs exert their influence on the progression of this condition. Figure 9A, B exhibit the immune cell infiltration percentages and Pearson correlations among immune cells for each sample. Notably, COQ2, MTPAP, NIPSNAP3A, FAM162A, MYO19, and NDUFS1 presented significant negative correlations with plasma cells, naive B cells, and naive CD4 T cells. Additionally, BCL2L11, GLDC, and CYP24A1 exhibited significant positive correlations with resting dendritic cells and resting mast cells, while displaying negative correlations with plasma cells (Fig. 9C). Patients with periodontitis displayed significantly higher proportions of naive B cells, memory B cells, plasma cells, follicular helper T cells, M1 macrophages, and neutrophils than healthy individuals. Conversely, the proportions of activated memory CD4 T cells, regulatory T cells (Tregs), activated NK cells, resting dendritic cells, activated dendritic cells, and resting mast cells were significantly lower in patients with periodontitis (Fig. 10). These findings suggest a close association between hub MERGs and host immune processes in periodontitis.

### Results of consensus cluster analysis based on hub MERGs

**Fig. 11:**
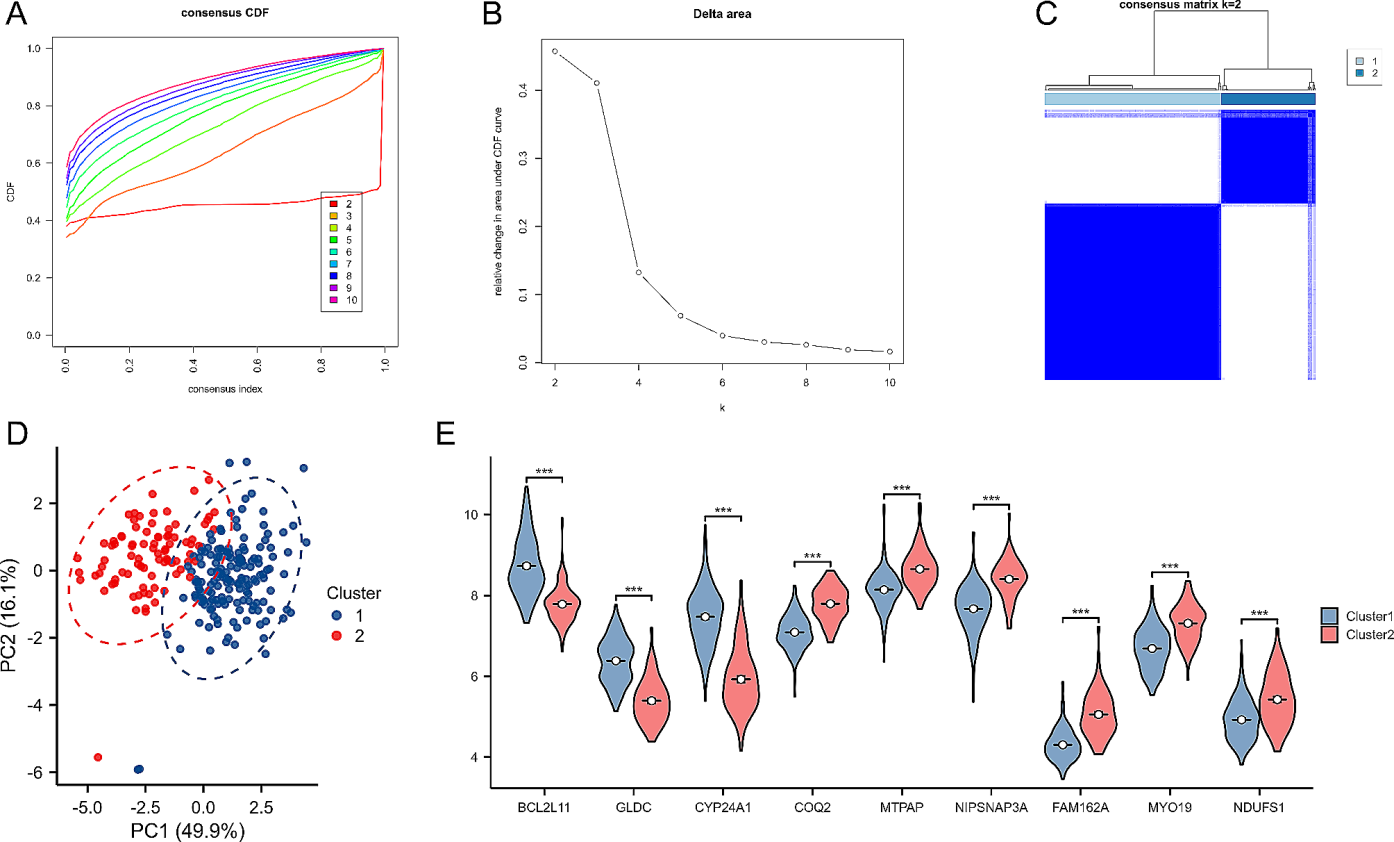
Identification of two distinct subtypes of hub MERG expression patterns in periodontitis. **A** Consensus clustering cumulative distribution function (CDF) for k = 2–9. **B** Relative change in the area under the CDF curve for k = 2–9. **C** Heatmap of the cooccurrence ratio matrix of periodontitis samples. **D** Principal component analysis of MERGs in subtype 1 and subtype 2. **E** Differences in the expression of hub MERGs between subtype 2 and subtype 1; ***P < 0.001

**Fig. 12:**
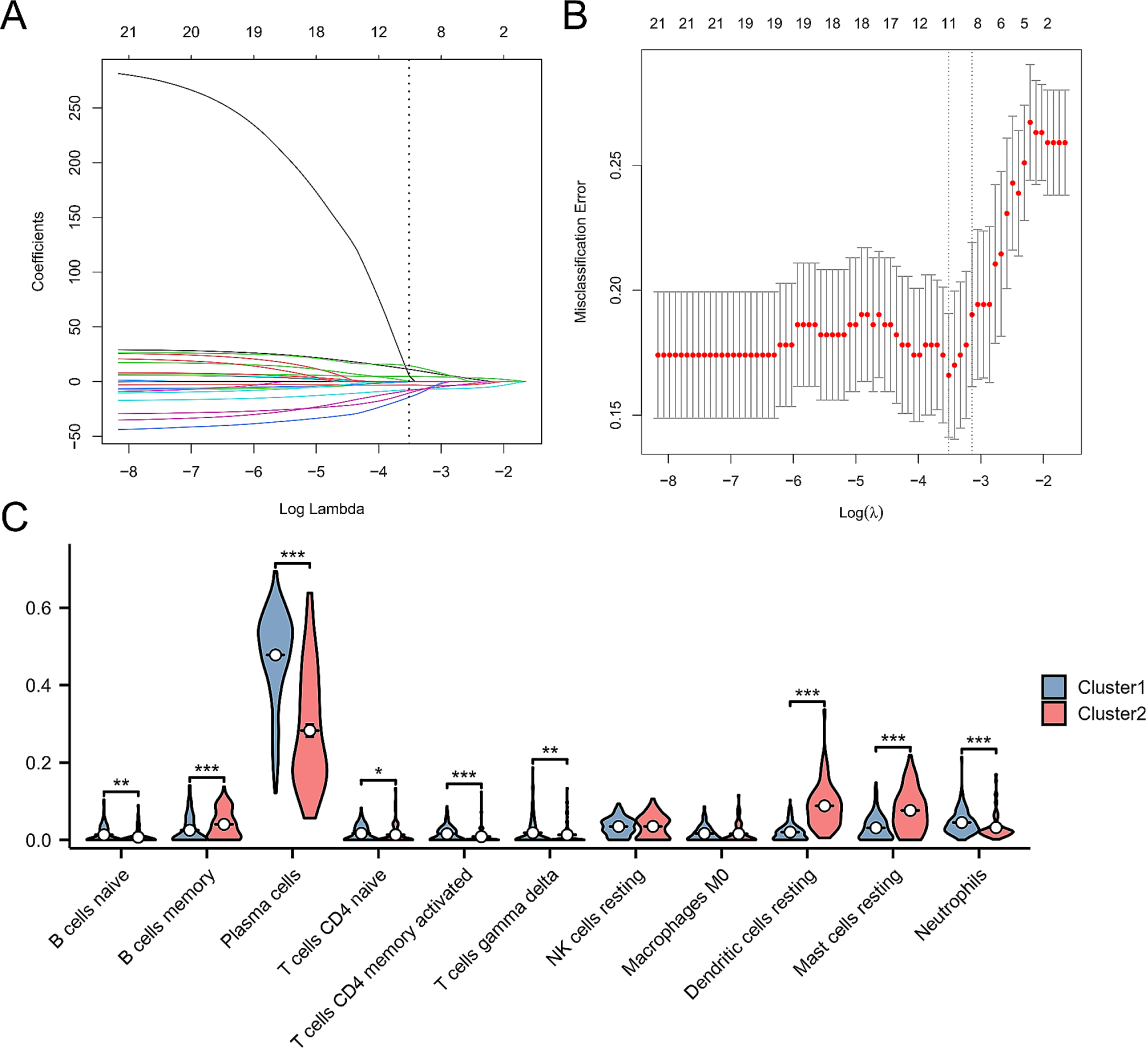
**A** LASSO coefficient profiles of 22 immune cell types. **B** Tenfold cross-validation was performed to identify the optimal tuning parameter (λ). **C** Expression differences in the filtered immune cells between subtype 2 and subtype 1; *P < 0.05, **P < 0.01, ***P < 0.001

To further analyze the expression of hub MERGs in periodontitis, this study used a consensus clustering algorithm to identify 247 periodontitis samples based on hub MERGs. The best number of clusters was found when k = 2 (Fig. 11A, B). The 247 periodontitis samples were divided into two clusters, cluster 1 (*n* = 153) and cluster 2 (*n* = 94) (Fig. 11C). Principal component analysis of the two clusters showed significant differences between them (Fig. 11D). Hub MERGs showed substantial differences in different periodontitis subtypes (Fig. 11E). Eleven critical immune cell types in periodontitis were screened by LASSO regression: naive B cells, memory B cells, plasma cells, naive CD4 T cells, activated memory CD4 T cells, gamma delta T cells, resting NK cells, M0 macrophages, resting dendritic cells, resting mast cells, and neutrophils (Fig. 12A, B). In different periodontitis subtypes (cluster 1 and cluster 2), naive B cells, memory B cells, plasma cells, activated memory CD4 T cells, gamma delta T cells, and gamma delta T cells were found. Memory, plasma cells, naive CD4 T cells, activated memory CD4 T cells, gamma delta T cells, resting dendritic cells, resting mast cells, and neutrophils showed significant differences (Fig. 12C). These results further elucidate the relevance of MitoEVs to the immune microenvironment in periodontitis.

### Results of the correlation analysis between hub MERG expression levels and probing depth in periodontitis

**Fig. 13:**
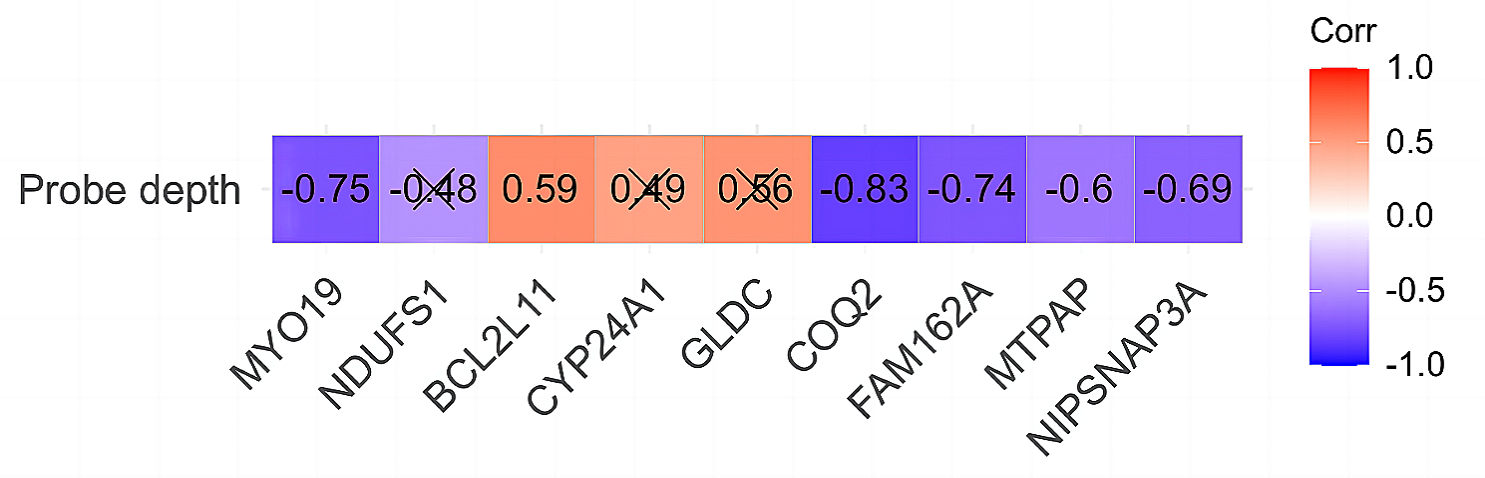
Pearson correlation analysis between hub MERG expression levels and probing depth. All correlation coefficients not indicating correlations are marked with “×”

In the GSE106090 dataset, BCL2L11 expression levels were significantly positively correlated with probing depth, and COQ2, MTPAP, NIPSNAP3A, FAM162A, and MYO19 expression levels were significantly negatively correlated with probing depth (Fig. 13).

## Discussion

The main features of periodontitis are inflammation caused by microorganisms in the soft tissues of the periodontium and progressive alveolar bone resorption. The MERGs screened in periodontitis in this study were BCL2L11, GLDC, CYP24A1, COQ2, MTPAP, NIPSNAP3A, FAM162A, MYO19, and NDUFS1. Through deep sequencing, YG et al. conducted an analysis of RNA samples derived from a mixture of gingival tissues obtained from both healthy individuals and patients with periodontitis. Their investigation identified GLDC as one of the prominently upregulated genes [33]. In a separate study by Li et al., the impact of *Enterococcus faecalis* OG1RF on the apoptosis of human cranial osteoblasts was explored. Notably, they observed an upregulation of pro-apoptotic BCL2L11 expression, implying the potential of BCL-2 family members as therapeutic targets for persistent periapical lesions [34]. Additionally, a study conducted by Chen et al. revealed CYP24A1 as a potential immunotherapy marker and target for periodontitis treatment [35]. Coenzyme Q, recognized for its redox-active lipid properties in facilitating electron transfer within the mitochondrial respiratory chain and functioning as an antioxidant in the plasma membrane, has been found to play a role in limiting lipid peroxidation and iron-induced cell death [36]. mtPAP is a mitochondrial poly (A) polymerase that mediates mRNA polyadenylation [37]. NIPSNAP3A is a novel protein with a thus far undefined function and is a member of the NIPSNAP family. Its mRNA has been detected in the cells of a variety of tissues, such as the adrenal gland, mammary gland, liver, skeletal muscle, uterine cervix, and myocardium, and it is particularly highly expressed in skeletal muscle [38]. FAM162A encodes a protein involved in various biological processes, such as the activation of cysteine-type endopeptidase activity in the apoptotic process, the cellular response to hypoxia, and the positive regulation of the release of cytochrome c from mitochondria [39]. Myo19 is a mitochondria-associated myosin that binds both mitochondria and actin [40]. It has been demonstrated that Myo19 facilitates the translocation of mitochondria to filamentous pseudopods in response to reactive oxygen species (ROS) while also serving as a link between mitochondria and cortical actin at the plasma membrane [41]. As the largest core subunit, NDUFS1 is essential in mitochondrial complex I function and stability. When NDUFS1 is mutated, the activity of mitochondrial complex I is reduced by approximately 80%. This leads to altered NADH homeostasis in complex I, resulting in tumorigenesis [42, 43]. Mitochondria undergo oxidative stress when stimulated by external factors, and NDUFS1 undergoes glutathione inactivation. Electron transfer in the respiratory chain cannot be completed. Thus, the body produces a large amount of ROS, which can activate multiple signaling pathways to regulate the expression of related genes. It is important to note that the mechanism of action outlined above is based on the general understanding of the function of these genes in the current literature. For periodontitis, the actual biological mechanisms may be more complex and include the interactions between these molecules and other cell signaling pathways and molecules. In addition, the specific environment of periodontal tissues, such as microbial composition and host immune response patterns, can also have a significant impact on how these molecules regulate periodontitis. Therefore, in practical research, it is necessary to explore their role in periodontitis through experimental methods such as gene expression analysis and protein function experiments.

In this investigation, we observed a substantial association between MERGs and diverse immune cell populations. Furthermore, we identified noteworthy discrepancies in both immune cell composition and MERG expression among distinct subtypes of periodontitis. It is widely acknowledged that periodontitis is characterized by an inflammatory cascade triggered by the excessive r。/ecruitment and accumulation of immune cells. The collection and activation of intrinsic immune cells in the oral environment constitute the transition from an inherent to an acquired immune response in periodontitis [44, 45]. At this stage, innate immune cells, such as dendritic cells (DCs), macrophages, and natural killer cells (NKs), predominate. Thereafter, with the appearance of antigen-presenting cells (APCs) such as DCs and macrophages, cells that dominate the acquired immune response, such as T cells and B cells, begin to dominate the immune response, constituting the developed immune response phase [46]. The last stage is characterized by advanced lesions, with irreversible destruction of adherent tissue and bone loss. This response involves osteoblasts, osteoclasts, and a variety of intrinsic and adaptive immune cells [47]. Various immune cells play different roles in different stages of periodontitis. Among these cells, neutrophils can inhibit the inflammatory response in chronic periodontitis by killing bacteria, releasing interleukin-10 (IL-10) and accelerating tissue destruction by promoting the inflammatory response through multiple pathways [48]. Monocyte macrophages can suppress inflammation, but M1-type macrophages can accelerate periodontitis progression [49]. Different subtypes of lymphocytes are also involved in the inflammatory process, and helper T cells (Th cells) 1, Th17, and memory B lymphocytes promote periodontal inflammation [49–51]. In contrast, regulatory T cells (Treg cells) and B10 cells can significantly inhibit the inflammatory response [52, 53]. It is essential to focus on the activation of various immune cells in the inflammatory response during the treatment of periodontitis to provide a guided immunomodulatory regimen to control the inflammatory response. Immune cells can release different types of EVs, which play essential roles in regulating immune responses and interactions between immune cells. For example, dendritic cells trigger an immune response by releasing exosomes that carry antigens and interact with other immune cells. In addition, immune cells such as T cells, B cells, and natural killer cells can also release signaling molecules such as cytokines and antibodies that regulate the immune response via EVs [54]. In addition, immune cell-derived EVs are involved in pathological processes, such as autoimmune regulation, immune tolerance, and immune dysregulation. Studies have shown that EVs released by immune cells can act as signal transducers in immune response pathways and regulate the activity and function of immune cells. EVs can also mediate the presentation and display of antigens by acting as antigen presenters. In addition, EVs can interact with other immune cells to promote the spread or limit the inflammatory response [55]. Overall, there are extensive interactions between extracellular vesicles and immune cells. Immune cells regulate immune responses, transmit signals, and mediate cellular interactions by releasing EVs.

There is a close interaction between immune cells and mitochondria. Mitochondria are intracellular organelles primarily responsible for energy production and the regulation of cellular metabolism. Recent studies have shown that mitochondria are not only a source of energy but also play an essential role in immune regulation and inflammatory processes [54]. First, mitochondria are involved in the activation and proliferation of immune cells. The functional and metabolic state of mitochondria is altered after immune cells are subjected to activation signals. By regulating metabolic pathways such as oxidative phosphorylation and glycolysis, mitochondria provide immune cells with the required energy and synthetic substances to support their activation and proliferation [56]. Second, mitochondria are considered signaling regulators of immune cells. Various important molecules are present in mitochondria, such as ROS, apoptosis-associated proteins, and mitochondrial DNA, which can play important regulatory roles in the immune response, either through the mitochondria themselves or after being released into the cytosol. For example, ROS released by mitochondria can act as signaling molecules to activate inflammatory responses, and mitochondrial DNA can be recognized as either “self” or “nonself” to trigger immune cell responses [57]. In addition, mitochondrial dysfunction is closely related to the onset and progression of immune-related diseases. Mitochondria can maintain their dynamic homeostasis through division, fusion, translocation, and autophagy [58]. Mitochondrial quality control includes three components: mitochondrial biogenesis, kinetics, and autophagy. Any one of the component disorders will lead to mitochondrial dysfunction, which in turn induces related diseases [59]. Several studies have found that mitochondrial dysfunction leads to the abnormal activation of immune cells, cell death, and immunoregulatory disorders, thereby promoting the development of inflammatory responses and autoimmune diseases [60]. In summary, mitochondria are essential in immune cells and regulate immune cell activation, signaling, and metabolism. In-depth study of the interaction mechanism between mitochondria and immune cells is of great significance for revealing the molecular mechanisms of immune regulation and developing therapeutic strategies for related diseases. A close relationship also exists between MitoEVs and immune cells, which play a vital role in immune regulation and inflammation. First, MitoEVs can be taken up by immune cells and function within recipient cells. Several studies have shown that after the uptake of MitoEVs, immune cells can utilize mitochondrial DNA and related molecules in Mito-EVs to activate mitochondria-associated immune responses, including type I interferon (IFN-I) production, inflammatory apoptosis, and immune response modulation [61]. In addition, MitoEVs modulate the function and activity of immune cells by regulating apoptotic and metabolic pathways [62]. Second, MitoEVs are closely related to immune inflammatory responses. It has been found that intracellular mitochondrial damage or overactivation increases the release of MitoEVs under inflammatory conditions. These MitoEVs can act as signaling molecules to deliver mitochondrial DNA and other mitochondria-associated molecules, thus playing an essential regulatory role in the inflammatory response [63]. For example, some studies have found that ingesting excess MitoEVs triggers inflammatory responses and apoptosis in immune cells, thereby exacerbating inflammatory lesions [64]. In conclusion, there is an interaction between MitoEVs and immune cells. Upon ingesting MitoEVs, immune cells can utilize mitochondrial DNA and other molecules to regulate immune responses and inflammatory reactions.

In summary, MitoEVs may have multiple roles in periodontitis, potentially involved in both the conduction of inflammatory signals and the repair of damaged tissues. Research into MitoEVs may provide new strategies to treat periodontitis, such as using engineered vesicles to alleviate inflammation or promote tissue regeneration. However, more research is still needed to determine the exact role of MREV in the pathogenesis of periodontitis and its potential for clinical application. This study also has some limitations. It is a bioinformatics analysis based on a public database, and the lack of experimental validation may bias the results. In addition, further research data are needed to test the reliability of the model in the future.

## Conclusion

The periodontitis classification model constructed based on hub MERGs shows excellent performance and can offer novel insights into the pathogenesis of periodontitis. The robust correlation between central hub MERGs and diverse immune cell populations, coupled with the substantial variations observed in immune cell profiles across different subtypes, helps elucidate the regulatory role of MitoEVs within the immune microenvironment of periodontitis. Future research should focus on elucidating the functional mechanisms of hub MERGs and exploring potential therapeutic interventions based on these findings.

## Data Availability

The data supporting this study’s findings are available from the corresponding author.

## Abbreviations

MitoEVs: Mitochondrial extracellular vesicles
EVs: Extracellular vesicles
MERGs: MitoEV-related genes
PDLSCs: Periodontal ligament stem cells
GEO: Gene expression omnibus
KEGG: Kyoto encyclopedia of genes and genomes
GO: Gene ontology
BP: Biological process
CC: Cellular component
MF: Molecular functions
ROC: Receiver operating characteristic
AUC: Area under the curve
BCL2L11: BCL2 Like 11
GLDC: Glycine Decarboxylase
CYP24A1: Cytochrome P450 Family 24 Subfamily A Member 1
COQ2: Coenzyme Q2, Polyprenyltransferase
MTPAP: Mitochondrial Poly(A) Polymerase
NIPSNAP3A: Nipsnap Homolog 3 A
FAM162A: Family With Sequence Similarity 162 Member A
MYO19: Myosin XIX
NDUFS1: NADH:Ubiquinone Oxidoreductase Core Subunit S1
